# Supplementary material for: Peripheral immune cell abundance differences link blood mitochondrial DNA copy number and Parkinson’s disease
Source: NPJ Parkinsons Dis. 2024 Nov 14;10:219. doi: 10.1038/s41531-024-00831-x (PMC11564539; doi:10.1038/s41531-024-00831-x)
Supplement: Supplementary file 1 — Supplementary Material [file 41531_2024_831_MOESM1_ESM.pdf]

Supplementary Notes

mitoCN

Homology Bias Adjustment

We generated bin annotation data with a bin size of 100 base pairs using the "createBins()" function. This data encompasses chromosome names, the positions of the first and last base pair within each bin, the percentage of characterized nucleotides (A, C, G, or T, i.e. non-N), and the GC content (percentage of C and G nucleotides among non-N nucleotides). Subsequently, we computed the average mappabilities per read bin using the "calculateMappability()" function. This involved employing a mappability file in bigWig format and the bigWigAverageOverBed binary. Specifically, we derived a 50-mer mappability file with 2 mismatches using GenMap (v1.3.0)[1] for the hg38 genome assembly. For hg19, we utilized the "wgEncodeCrgMapabilityAlign50mer.bigWig" file from the ENCODE's download section of the UCSC Genome Browser. The bigWigAverageOverBed binary can be downloaded from the UCSC Genome Browser's Other Utilities section. Additionally, we determined the percentage overlap between the generated bins and ENCODE's Blacklisted Regions via the "calculateBlacklist()" function. Any read bins with mappability below 100% or overlap with blacklist regions exceeding 0% were excluded. These analyses were performed using R software (version 4.3.1) with the QDNAseq[2] R package (version 1.38.0).

GC Bias Adjustment

After selecting read bins with mappability = 100%, the GC content range on mtDNA spans from 30% to 60%. We segmented this range into six groups, each with 5% intervals, denoted as g_i (i = 1, 2, …, 6). Let $\mathcal{M}_i$ and $\mathcal{A}_i$ represent the collection of 100bp read bins B, we use in the mitochondrial and autosomal genomes, respectively, falling within the GC content range g_i. Write $|\mathcal{M}_i| = m_i$ and $|\mathcal{A}_i| = a_i$ for the number of read bins in these collections. We use $m_+ = \sum_{i=1}^{6} m_i = 20$ mitochondrial bins with differing GC content, all with 100% mappability. We then randomly select 20 bins in each chromosome that match the characteristics of the mitochondrial bins in terms of GC content and mappability, repeating this process

k times. Using bins from only one autosome, $a_i = km_i$ so $a_+ = km_+ = 20k$, while using bins from all 22 autosomes, $a_+ = 440k$.

$M(B)$ is the number of reads with 3' end in the mitochondrial bin B while $A(B)$ is the number of reads with 3' end in the autosomal bin B. Assume that for $B \in \mathcal{M}_i$, we have $M(B) \sim Poisson(\mu Np\beta_i)$, while for $B \in \mathcal{A}_i$, we assume that $A(B) \sim Poisson(2Np\beta_i)$.

The log-likelihood generated by the bin counts under these assumptions is,

$$l = \Sigma_i \left[\Sigma_{B\in\mathcal{M}_i} \{-\mu Np\beta_i + M(B)log\,(\mu Np\beta_i)\} + \Sigma_{B\in\mathcal{A}_i} \{-2Np\beta_i + A(B)log\,(2Np\beta_i)\}\right]$$

which when we write $M_i = \Sigma_{B\in\mathcal{M}_i} M(B)$ and $A_i = \Sigma_{B\in\mathcal{A}_i} A(B)$, simplifies to

$$l = \Sigma_i \left[-\mu Npm_i\beta_i + M_i log(\mu Np\beta_i) - 2Npa_i\beta_i + A_i log(2Np\beta_i)\right].$$

We differentiate this with respect to μ and the $\{\beta_i\}$ and equate the derivatives to 0, getting estimating equations for

$$\mu = \frac{\Sigma_i \Sigma_{B\in\mathcal{M}_i} M(B)}{Np\Sigma_i m_i\beta_i} = \frac{M}{Np\Sigma_i m_i\beta_i} \ldots\ldots (1)$$

$$\beta_i = \frac{T_i}{Np(\mu m_i + 2a_i)} \ldots\ldots\ldots\ldots\ldots (2)$$

where M is the total number of reads mapping to mtDNA, $T_i = \Sigma_{B\in\mathcal{M}_i} M(B) + \Sigma_{B\in\mathcal{A}_i} A(B)$.

Correction in the Gupta et al., 2023

We have identified an error in the Gupta et al., 2023 study[3], where the effect sizes of the genome-wide association studies (GWASs) were incorrectly signed for the reference allele listed. Consequently, this error has impacted several interpretations in the paper, such as Figure 1E, and the direction of all Mendelian randomization (MR) plots (Figure 1G, 1H, EDF4G, EDF4H, and EDF6). We have communicated our concerns to the authors, and they have addressed this issue by updating the GWAS Catalog summary statistics on April 5, 2024, by swapping the allele listed in the "effect allele" and have submitted a corresponding correction to Nature. The GWAS summary statistics utilized in this study were those post-correction.

Furthermore, it's important to note that the output file from the mtSwirl v2.5_MongoSwirl_Single pipeline lacks results for both mitochondrial DNA copy number (mtDNA-CN) and mean nuclear DNA (nucDNA) coverage (https://github.com/rahulg603/mtSwirl/issues). Users are required to modify the pipeline by adding commands for nucDNA calculation before applying it on Terra and calculate mtDNA-CN independently. This may impact the reproducibility of the tool. In contrast, mitoCN is a single-command tool and is more user-friendly.

Immune Cell Distributions and Parkinson's Disease Risk in African Ancestry

In the AMP PD dataset, 109 individuals are identified with genetically defined African ancestry. Among these, only 48 have baseline whole blood bulk RNA-sequencing data available for estimating cell composition, which includes 21 PD cases and 27 controls. We evaluated whether the associations of lymphocyte and neutrophil levels, as well as the neutrophil-to-lymphocyte ratio (NLR), with PD risk in the African ancestry group differ from those observed in the broader multi-ancestry cohort. Although the effect size directions are consistent with those in the multi-ancestry cohort (Supplementary Table S9), the limited sample size likely impeded the detection of significant differences in immune cell distributions between PD cases and controls.

In the UK Biobank (UKB), out of 5,953 individuals with genetically defined African ancestry, only 28 have been diagnosed with PD. We assessed the associations between PD risk and immune cell abundance using the same methods applied to the entire UKB cohort and found no significant associations, likely due to the small number of PD cases (Supplementary Table S9). Notably, although no significant associations were found, the directions of effects on platelet count (African: beta = 18.774, $p = 0.5$, multi-ancestry: beta = -3.132, $p = 0.0006$) and plateletcrit (African: beta = 0.015, $p = 0.5$, multi-ancestry: beta = -0.003, $p < 0.0001$) in individuals of African ancestry differed from those in multi-ancestry. This discrepancy may account for the contradictory findings reported by Müller-Nedebock et al. (2022)[4] compared to other PD studies. Further research is necessary to elucidate the role of platelets in PD across different ancestries.

Overall, the small number of PD cases in both datasets limits our ability to demonstrate the associations between immune cell distributions and PD risk among individuals of African ancestry. The analysis scripts are available at https://github.com/bahlolab/mitoCN/tree/main/scripts/revision.

Supplementary References

1. Pockrandt, C., Alzamel, M., Iliopoulos, C. S. & Reinert, K. GenMap: Ultra-fast computation of genome mappability. *Bioinformatics* **36**, (2020).
2. Scheinin, I. *et al.* DNA copy number analysis of fresh and formalin-fixed specimens by shallow whole-genome sequencing with identification and exclusion of problematic regions in the genome assembly. *Genome Res* **24**, (2014).
3. Gupta, R. *et al.* Nuclear genetic control of mtDNA copy number and heteroplasmy in humans. *Nature* **620**, (2023).
4. Müller-Nedebock, A. C. *et al.* Increased blood-derived mitochondrial DNA copy number in African ancestry individuals with Parkinson's disease. *Parkinsonism Relat Disord* **101**, (2022).

Supplementary Figures

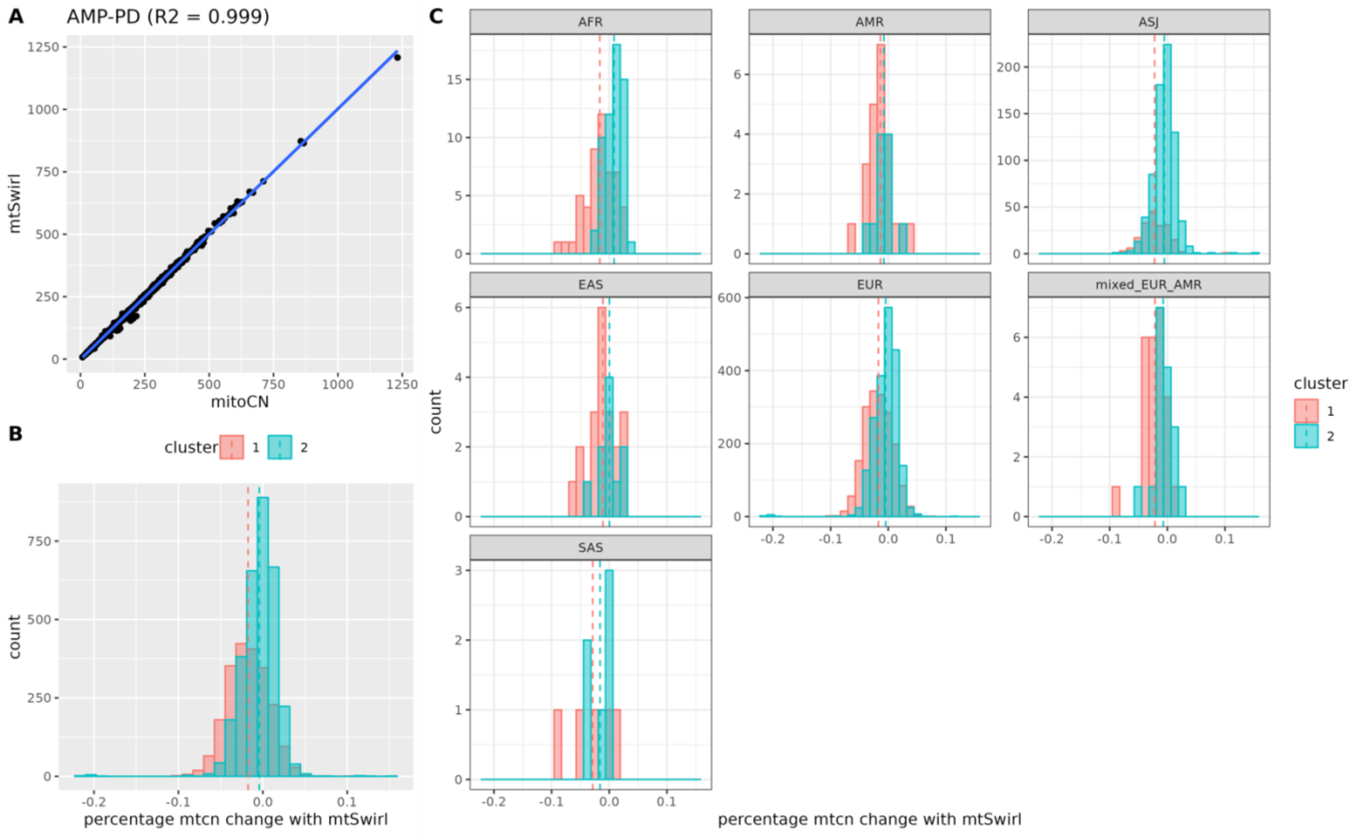

Supplementary Fig. S1 Comparison of estimates from mtSwirl and mitoCN.

A, scatter plot illustrates a high concordance in mtDNA-CN estimates between mitoCN and mtSwirl (R^2 = 0.999, p <2.2e-16); **B,** histogram shows the percent change in mtDNA-CN estimated from mitoCN and mtSwirl. The average percent change for cluster 1 is -1.8%, and for cluster 2, it is -0.4%; **C,** percent change in mtDNA-CN estimated using mitoCN versus mtSwirl, grouped by inferred nuclear ancestry. The superpopulation groups that can be inferred include AFR, AMR, ASJ, EAS, EUR, SAS, for African, American, Ashkenazi Jewish, east Asian, European, and south Asian, respectively. Cluster 1 is referred to as "platelet-depleted blood samples", while cluster 2 is referred to as "platelet-abundant blood samples".

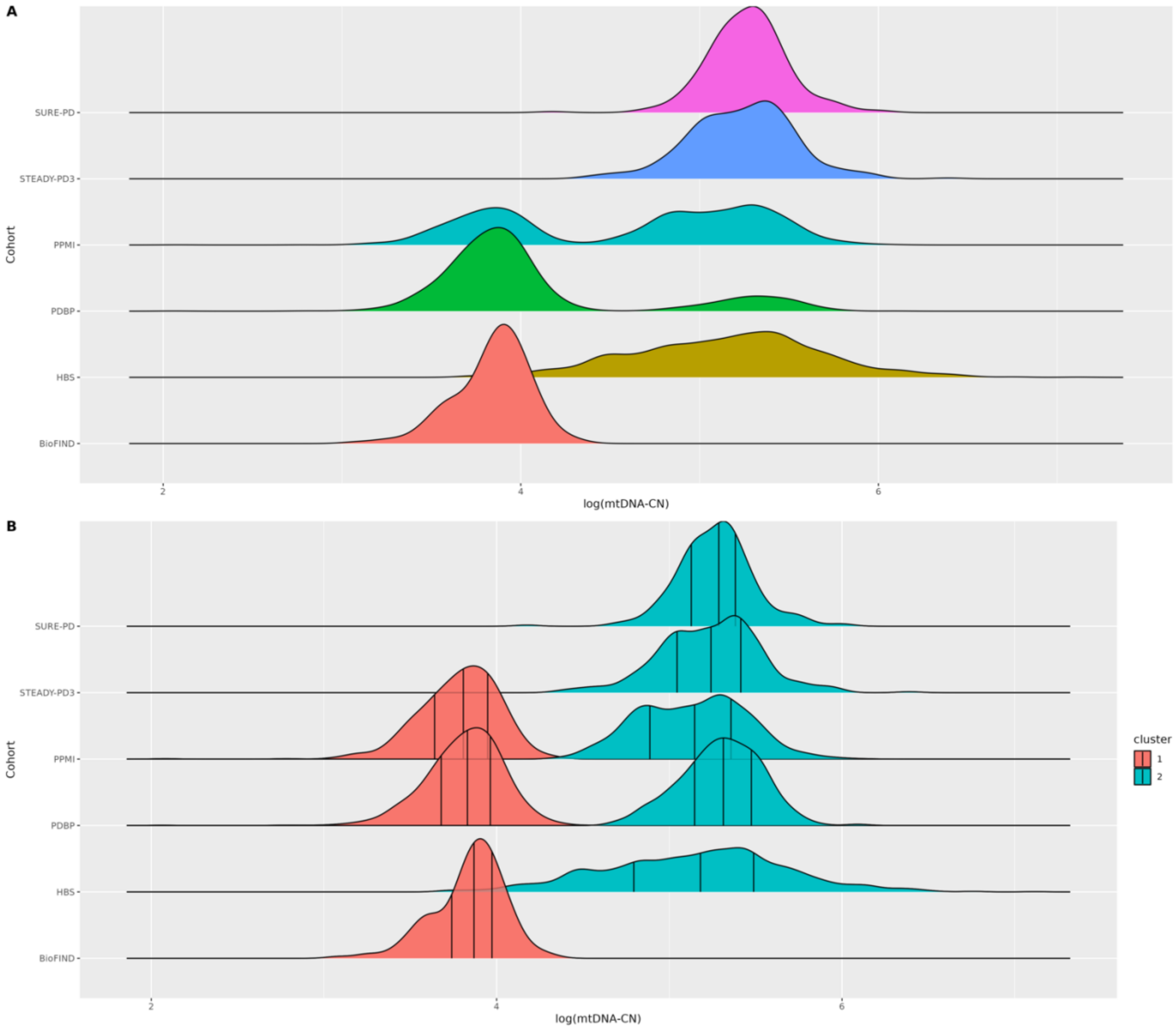

Supplementary Fig. S2 Log scaled mtDNA-CN distribution in AMP PD cohorts.

A, two distinct distributions in the mtDNA-CN estimates, suggesting that DNA samples were extracted from two types of blood samples; **B,** two clusters classified using Gaussian mixture model.

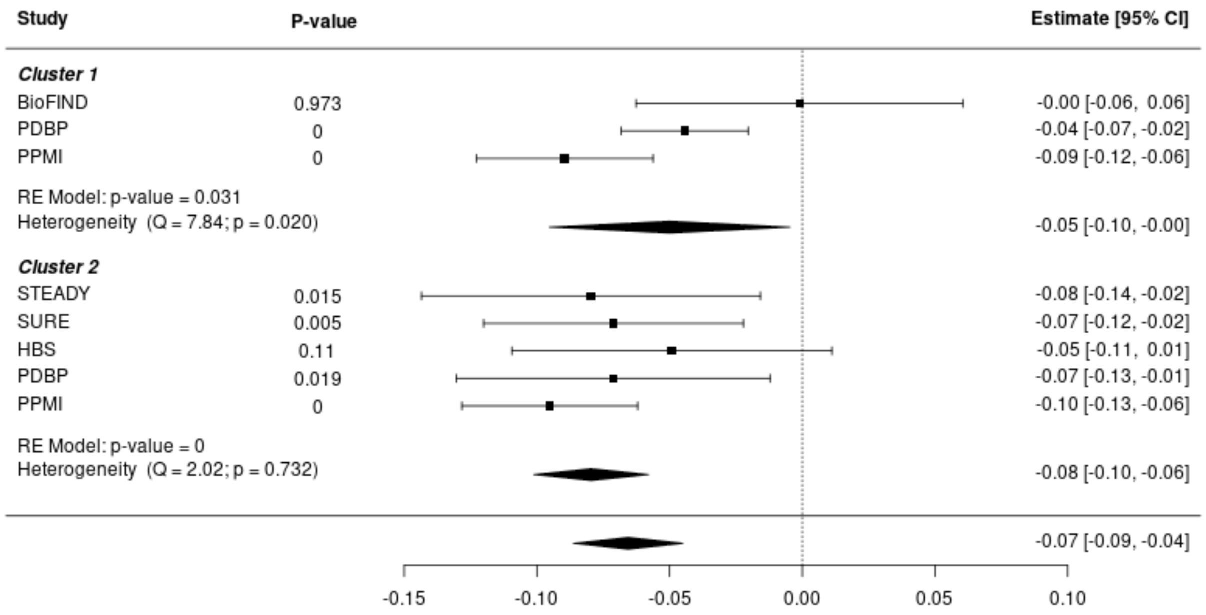

Supplementary Fig. S3 Meta-analysis of association tests between blood mtDNA-CN and sex.

Both blood sample types confirmed the association between sex and mtDNA-CN, indicating that mtDNA-CN tends to be lower in males compared to females. The absolute effect size estimated from cluster 2 (beta = -0.08, p <0.0001), which comprises platelet-abundant samples, is greater than that of cluster 1 (beta = -0.05, p = 0.03). Additionally, the variability of estimate from cluster 2 (95% CI [-0.09, -0.04]) is smaller than cluster 1 (95% CI [-0.10, 0]).

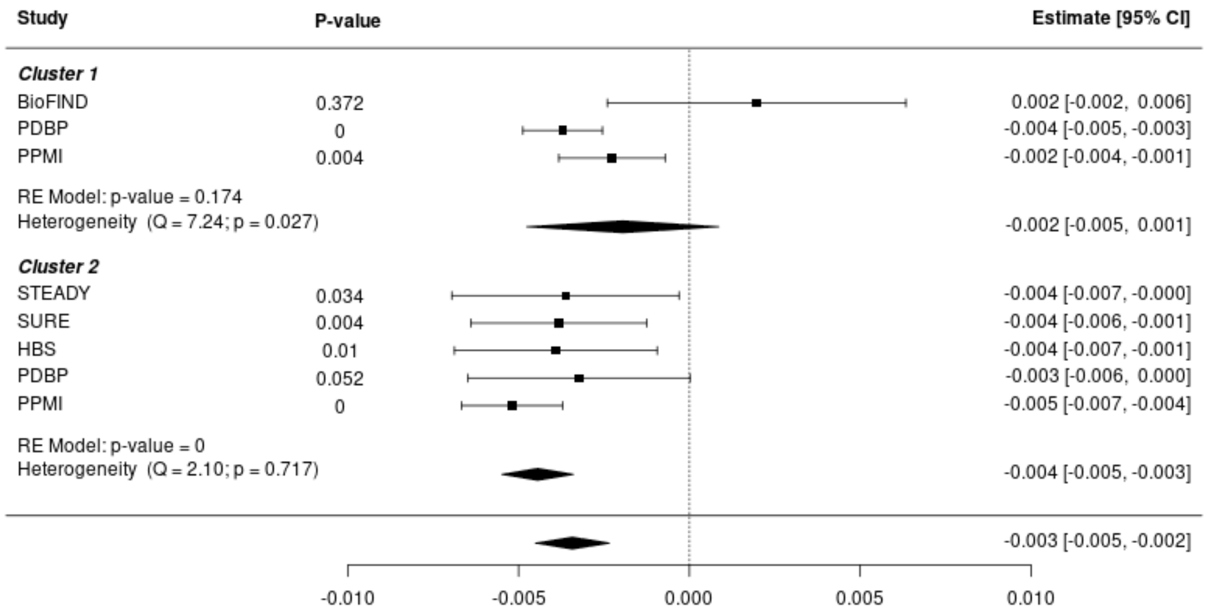

Supplementary Fig. S4 Meta-analysis of association tests between blood mtDNA-CN and age.

For cluster 2, the meta-analysis results indicate a significant decline in mtDNA-CN with age (beta = -0.004, p <0.0001). However, the association is not significant in cluster 1, mainly due to the heterogeneity of the BioFIND cohort. Upon excluding the BioFIND cohort, there is no evidence of heterogeneity between the PPMI and PDBP cohorts (Q = 2.12, p = 0.15), and the association becomes significant (beta = -0.003, p <0.0001).

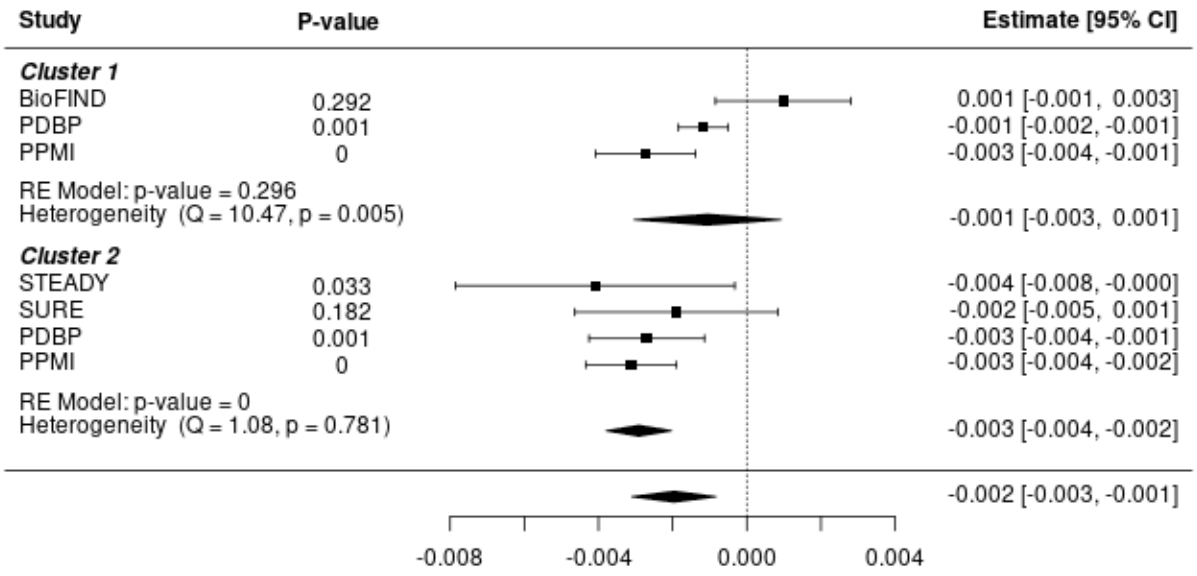

Supplementary Fig. S5 Meta-analysis of association tests between blood mtDNA-CN and motor examination (MDS-UPDRS III).

In cluster 2, the meta-analysis results reveal a significant correlation, indicating that lower mtDNA-CN is associated with more severe motor symptoms (beta = -0.003, p <0.0001). However, in cluster 1, this association lacks significance, mainly due to the heterogeneity from the BioFIND cohort. After excluding the BioFIND cohort, no significant heterogeneity is detected between the PPMI and PDBP cohorts (Q = 1.08, p = 0.78), and the association becomes significant (beta = -0.003, p <0.0001).

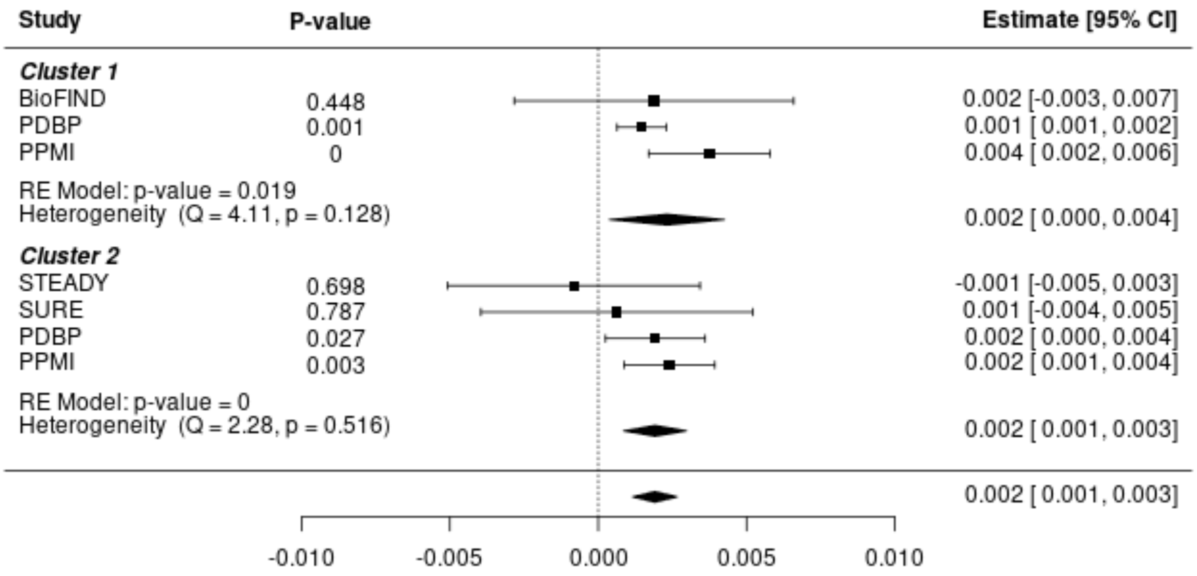

Supplementary Fig. S6 Meta-analysis of association tests between mtDNA-CN and activities of daily living (ADL).

Both clusters validate the correlation between ADL and mtDNA-CN, suggesting that reduced levels of mtDNA-CN are linked to impaired performance in daily activities. The effect size estimated from both clusters is 0.002. Moreover, the variability of the estimate from cluster 2 (95% CI [0.001, 0.003]) is narrower compared to cluster 1 (95% CI [0, 0.004]), indicating greater precision, and it is also more statistically significant.

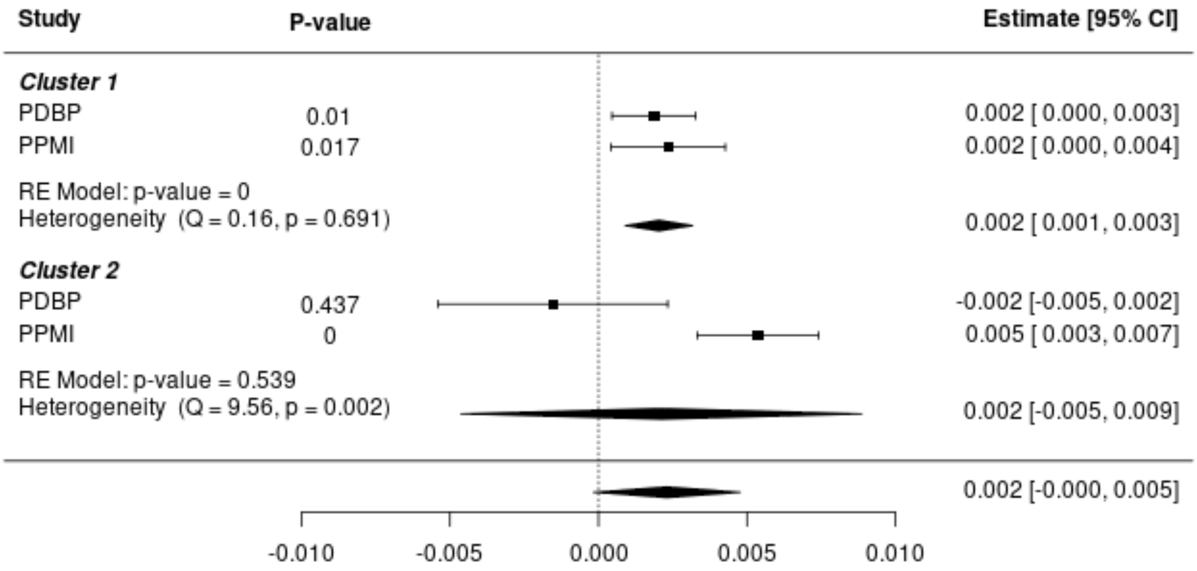

Supplementary Fig. S7 Meta-analysis of association tests between mtDNA-CN and olfactory impairment (UPSIT).

The olfactory bulb has been identified as one of the primary regions where PD is thought to initiate, and the UPSIT score has been linked to the severity of PD. In cluster 1, the meta-analysis reveals a significant association between lower mtDNA-CN and olfactory dysfunction (beta = 0.002, p <0.0001). However, in cluster 2, this association lacks significance, likely attributable to the heterogeneity of the PDBP and the PPMI cohorts (Q = 9.56, p = 0.002). Notably, the PDBP cohort in cluster 2 exhibits significant differences from the other cohorts. Focusing solely on the PPMI cohort within cluster 2, we observe a larger effect size compared to cluster 1, possibly due to mtDNA enrichment from platelets.

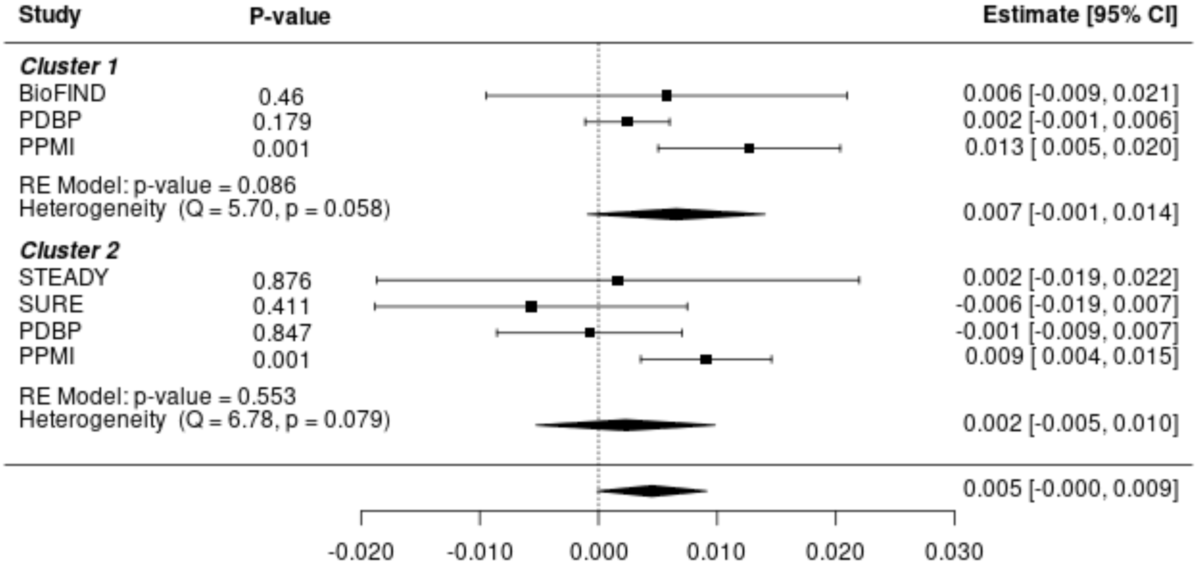

Supplementary Fig. S8 Meta-analysis of association tests between mtDNA-CN and cognitive impairment (MoCA).

Through cohort analysis, the association between mtDNA-CN and cognitive impairment was solely identified within the PPMI cohort across both clusters, while not observed in other cohorts. The meta-analysis results from both clusters indicate no significant associations.

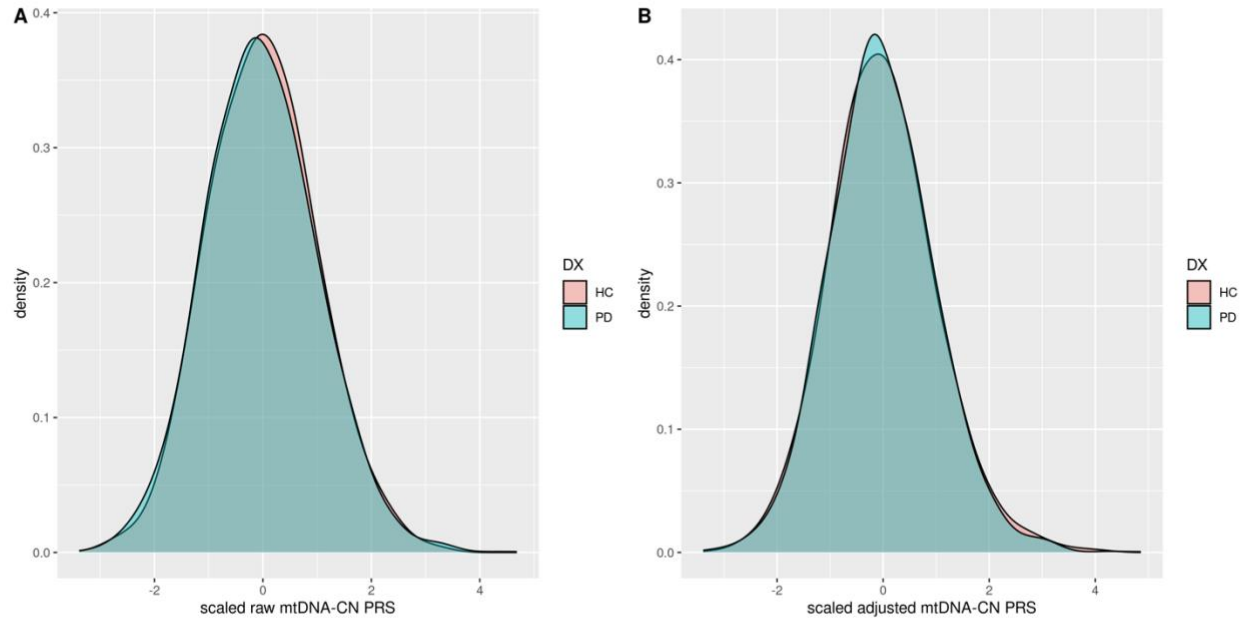

Supplementary Fig. S9 Density plots of mtDNA-CN PRSs in healthy controls (HC) and individuals diagnosed with PD.

A, scaled raw mtDNA-CN PRS in HC and PD; **B,** scaled adjusted mtDNA-CN PRS in HC and PD.

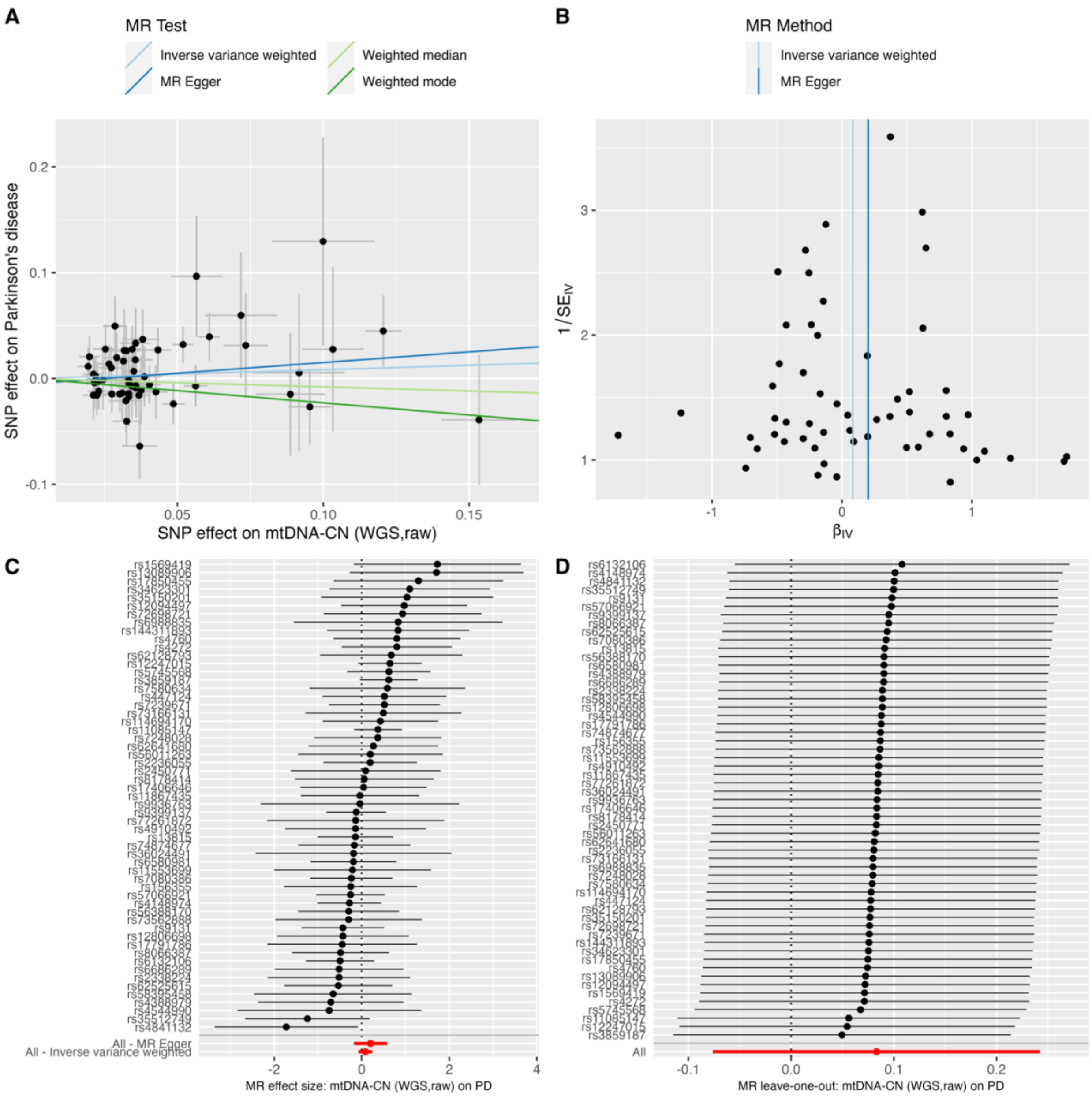

Supplementary Fig. S10 Mendelian Randomization analysis of mtDNA-CN (WGS, raw) on PD.

A, scatter plots showing the causal association; **B,** funnel plots; **C,** single SNP effect size plot; **D,** leave-one-out plot.

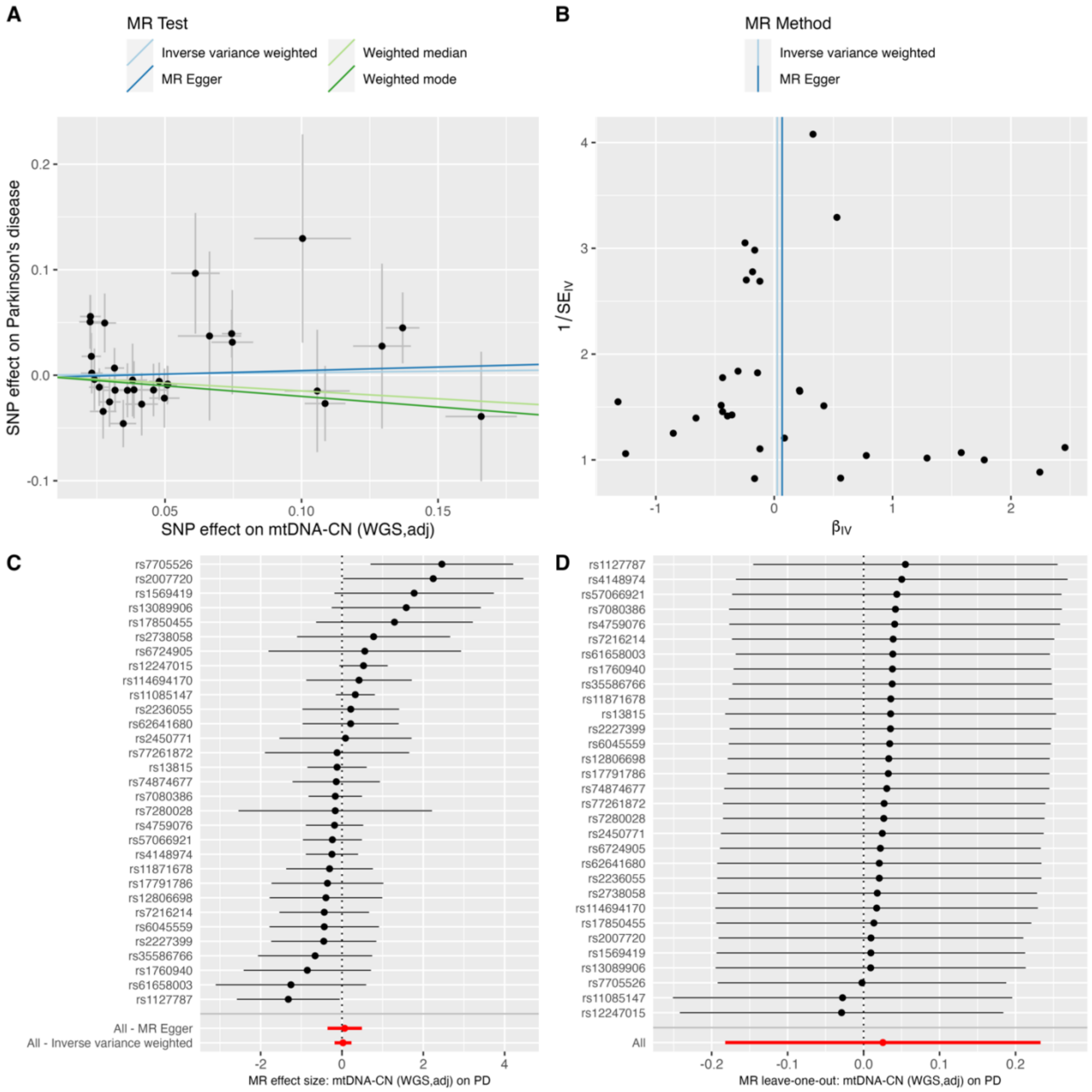

Supplementary Fig. S11 Mendelian Randomization analysis of mtDNA-CN (WGS, adjusted) on PD. **A,** scatter plots showing the causal association; **B,** funnel plots; **C,** single SNP effect size plot; **D,** leave-one-out plot.

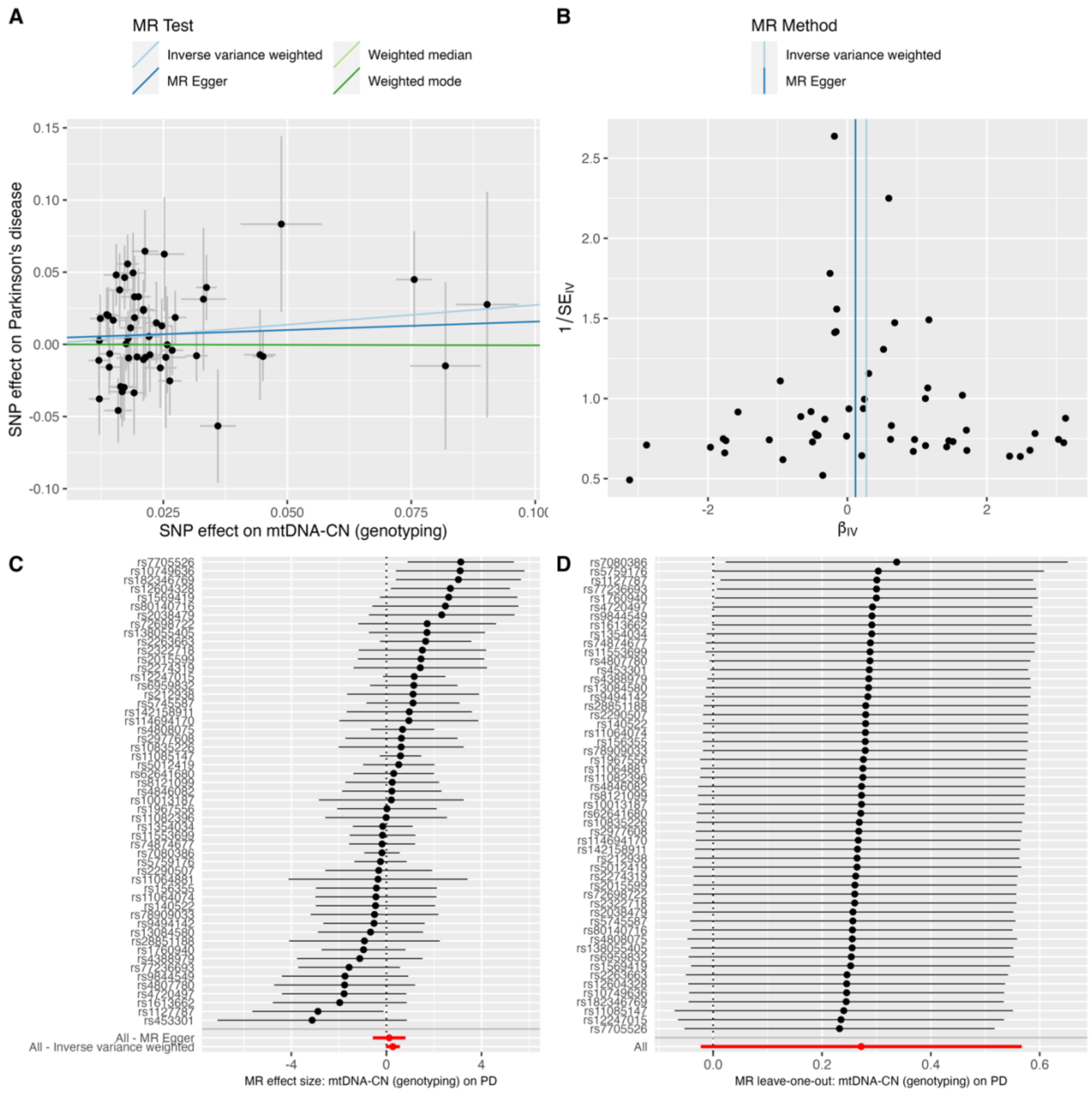

Supplementary Fig. S12 Mendelian Randomization analysis of mtDNA-CN (genotyping) on PD.

A, scatter plots showing the causal association; **B,** funnel plots; **C,** single SNP effect size plot; **D,** leave-one-out plot.

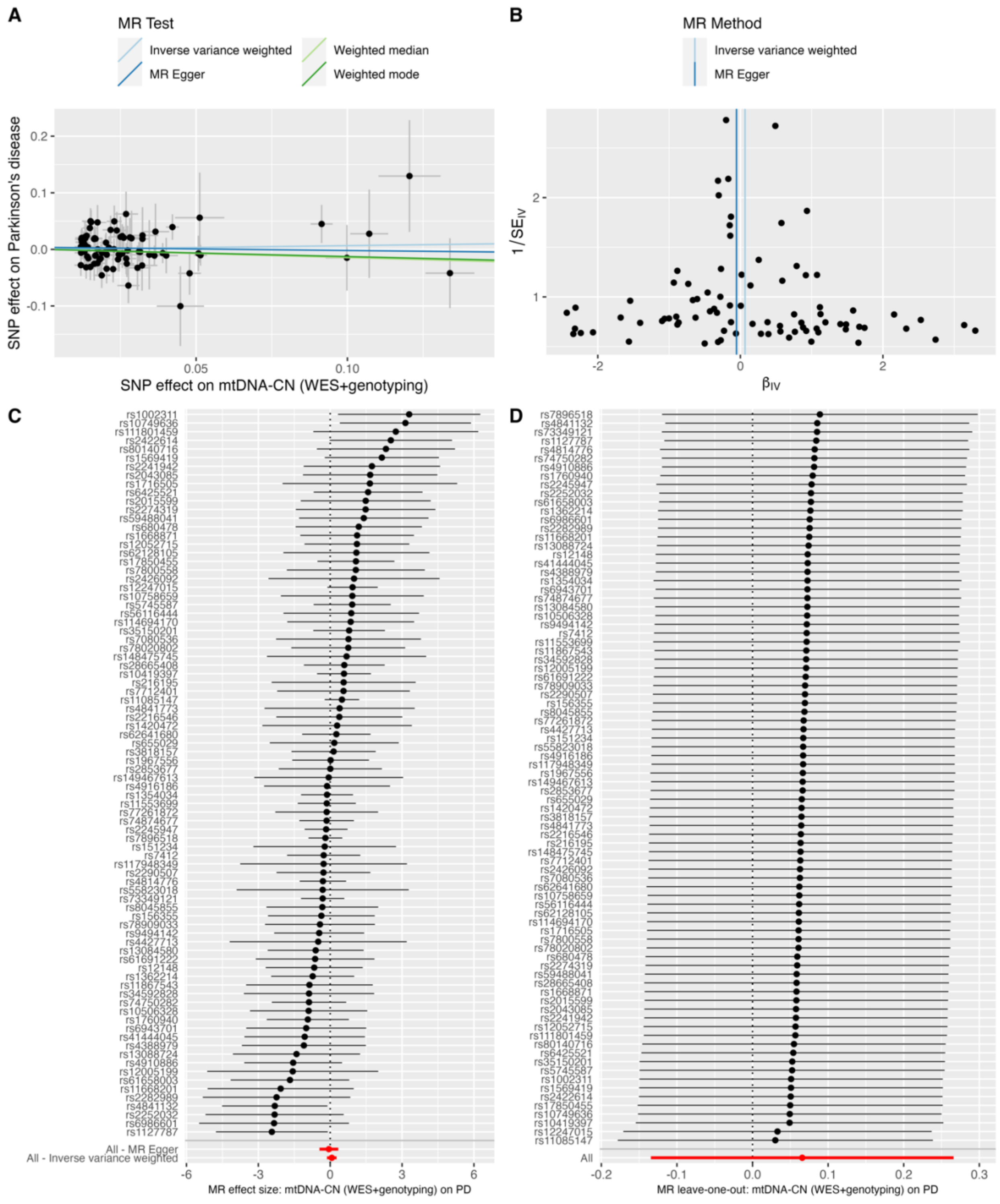

Supplementary Fig. S13 Mendelian Randomization analysis of mtDNA-CN (WES + genotyping) on PD.

A, scatter plots showing the causal association; **B,** funnel plots; **C,** single SNP effect size plot; **D,** leave-one-out plot.

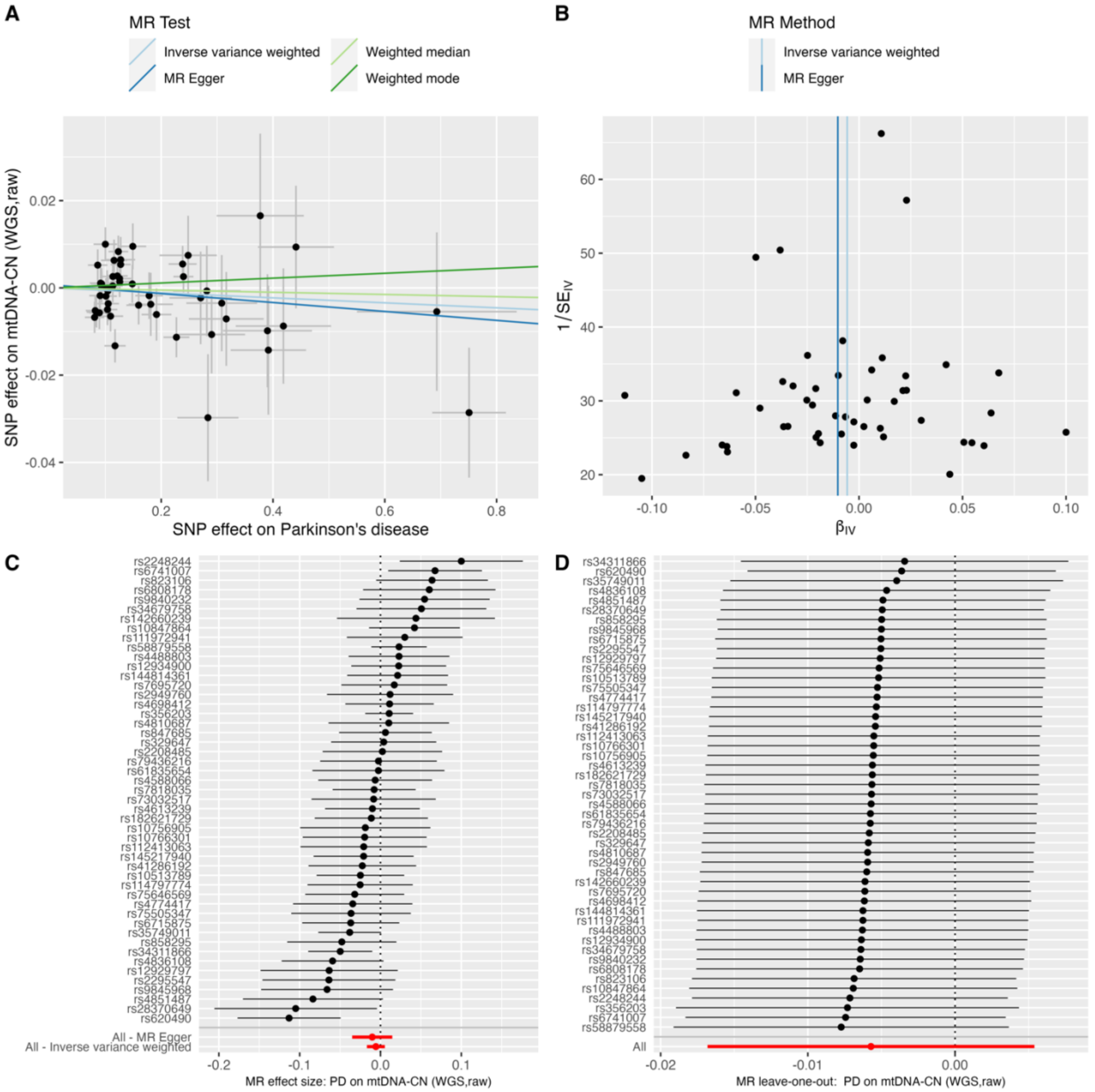

Supplementary Fig. S14 Mendelian Randomization analysis of PD on mtDNA-CN (WGS, raw).

A, scatter plots showing the causal association; **B,** funnel plots; **C,** single SNP effect size plot; **D,** leave-one-out plot.

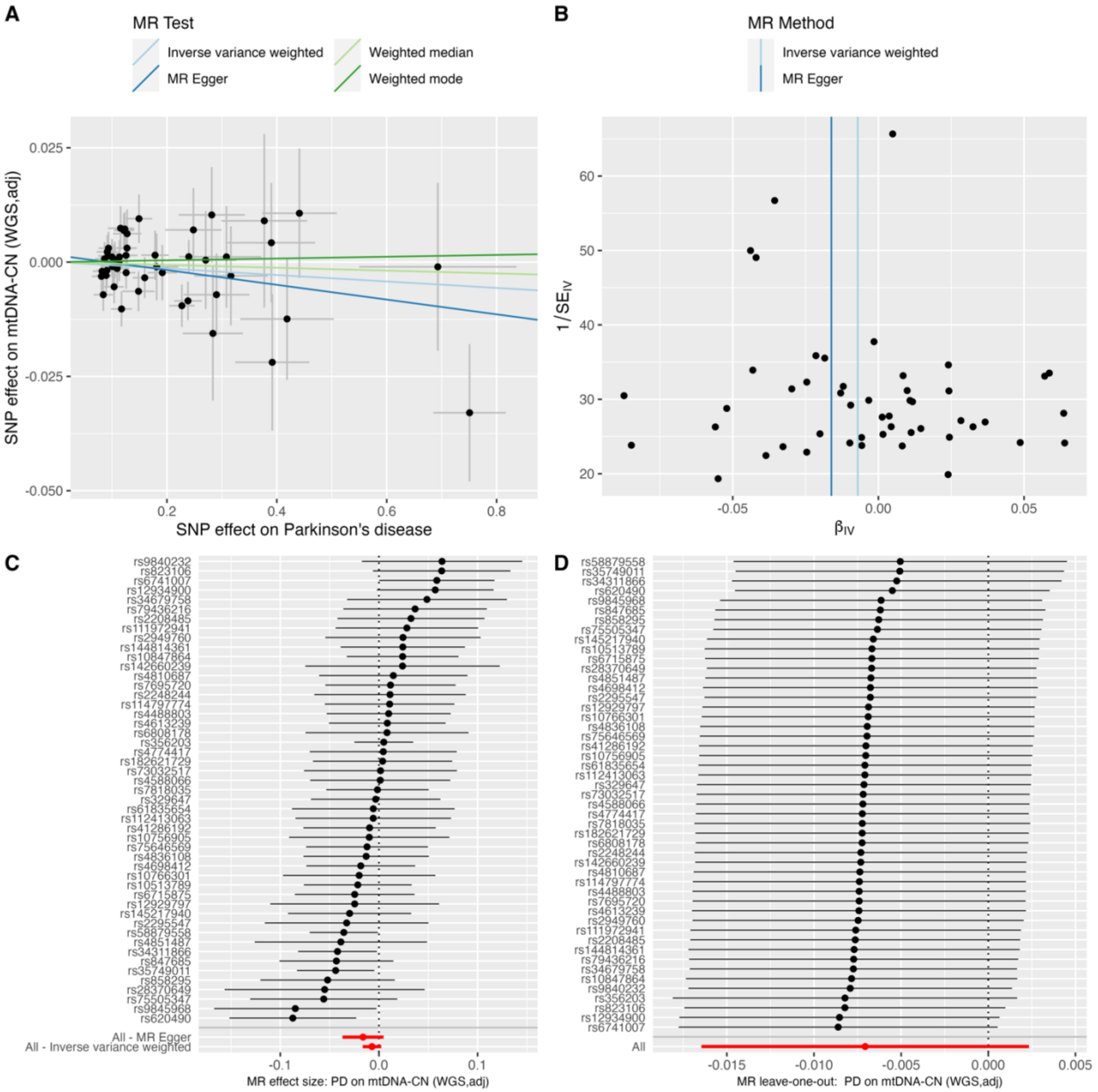

Supplementary Fig. S15 Mendelian Randomization analysis of PD on mtDNA-CN (WGS, adjusted).

A, scatter plots showing the causal association; **B,** funnel plots; **C,** single SNP effect size plot; **D,** leave-one-out plot.

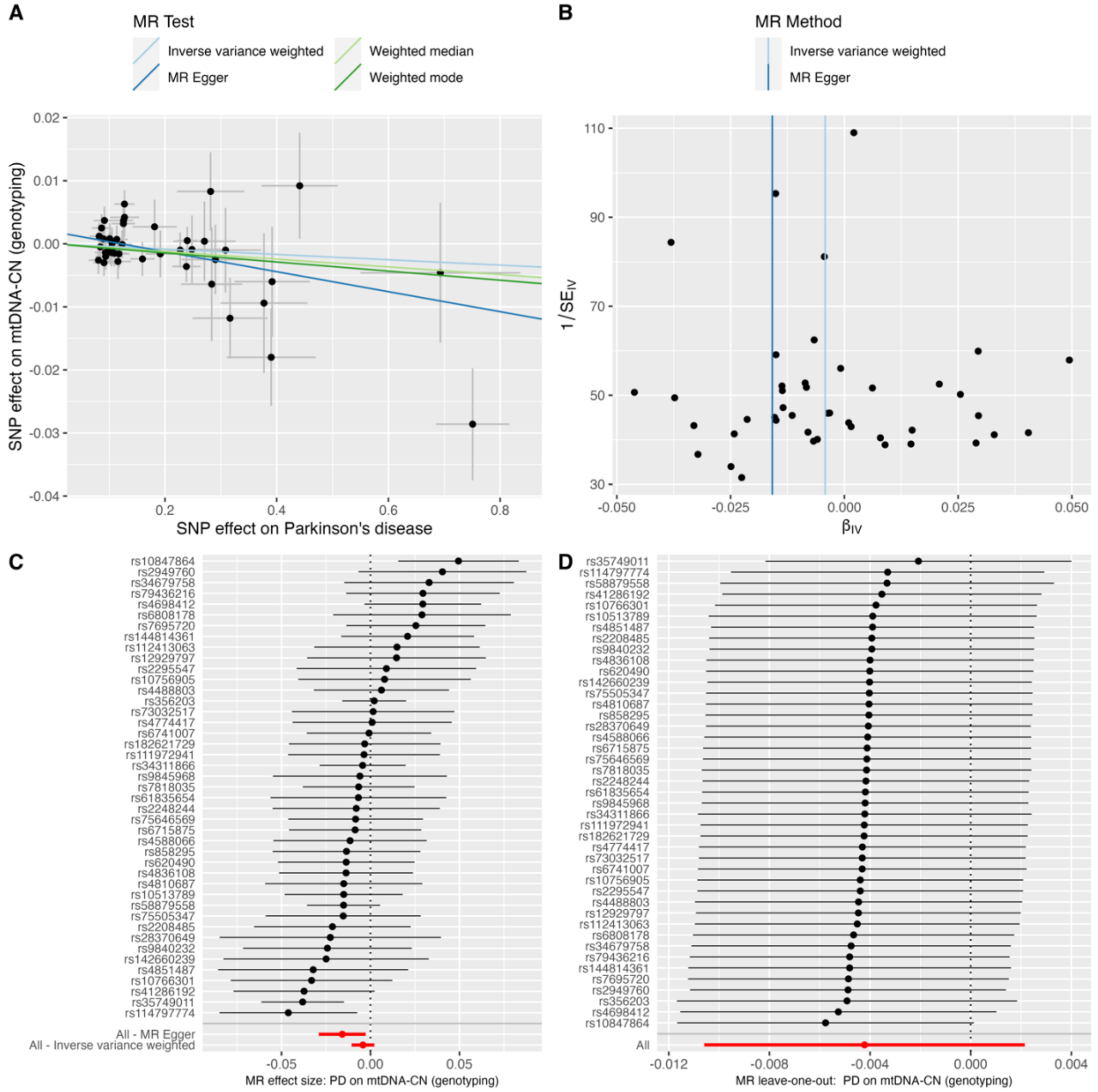

Supplementary Fig. S16 Mendelian Randomization analysis of PD on mtDNA-CN (genotyping).

A, scatter plots showing the causal association; **B,** funnel plots; **C,** single SNP effect size plot; **D,** leave-one-out plot.

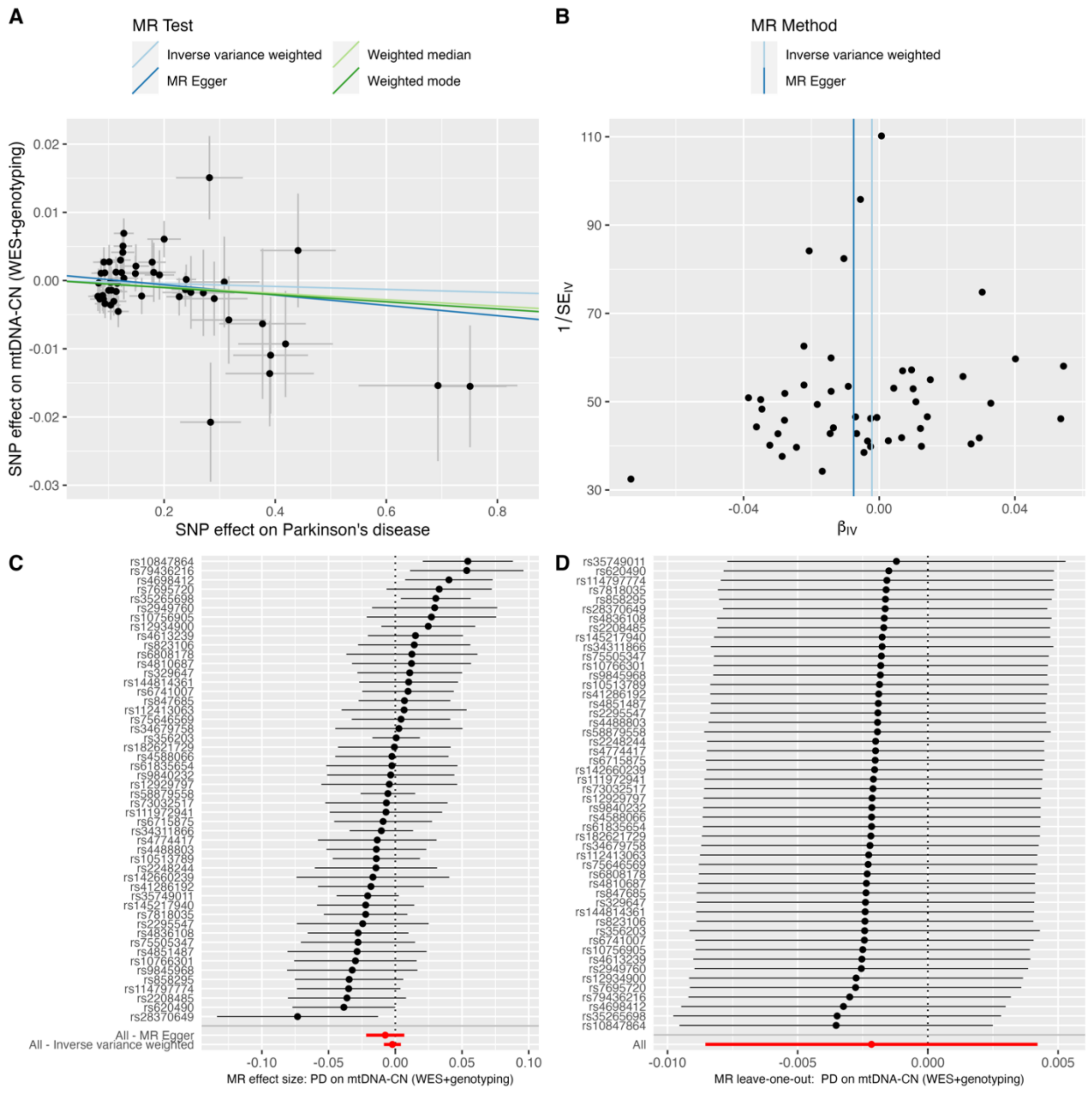

Supplementary Fig. S17 Mendelian Randomization analysis of PD on mtDNA-CN (WES + genotyping).

A, scatter plots showing the causal association; **B,** funnel plots; **C,** single SNP effect size plot; **D,** leave-one-out plot.

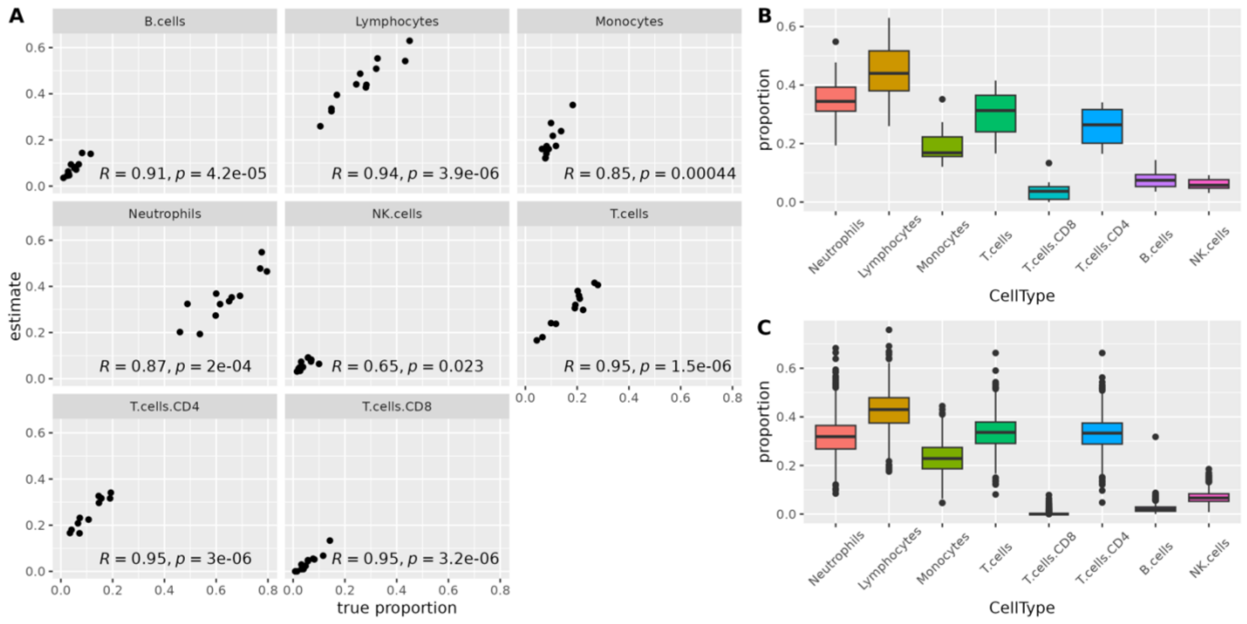

Supplementary Fig. S18 Validation of CIBERSORTx cell type proportion estimates.

A, Pearson correlation tests comparing estimated cell type proportions with ground truth proportions; **B,** boxplot displaying the range of estimated proportions for the validation cohort, consisting of whole blood bulk RNA-seq data from 12 healthy adults, in each of the eight major cell types. Center line, median; box limits, upper and lower quartiles; whiskers, 1.5x interquartile range; points, outliers; **C,** boxplot illustrating the range of estimated proportions for the bulk RNA-seq data from healthy controls in the AMP PD data. Lymphocyte proportions were derived with the cumulative proportions of T cells, B cells, natural killer (NK) cells.

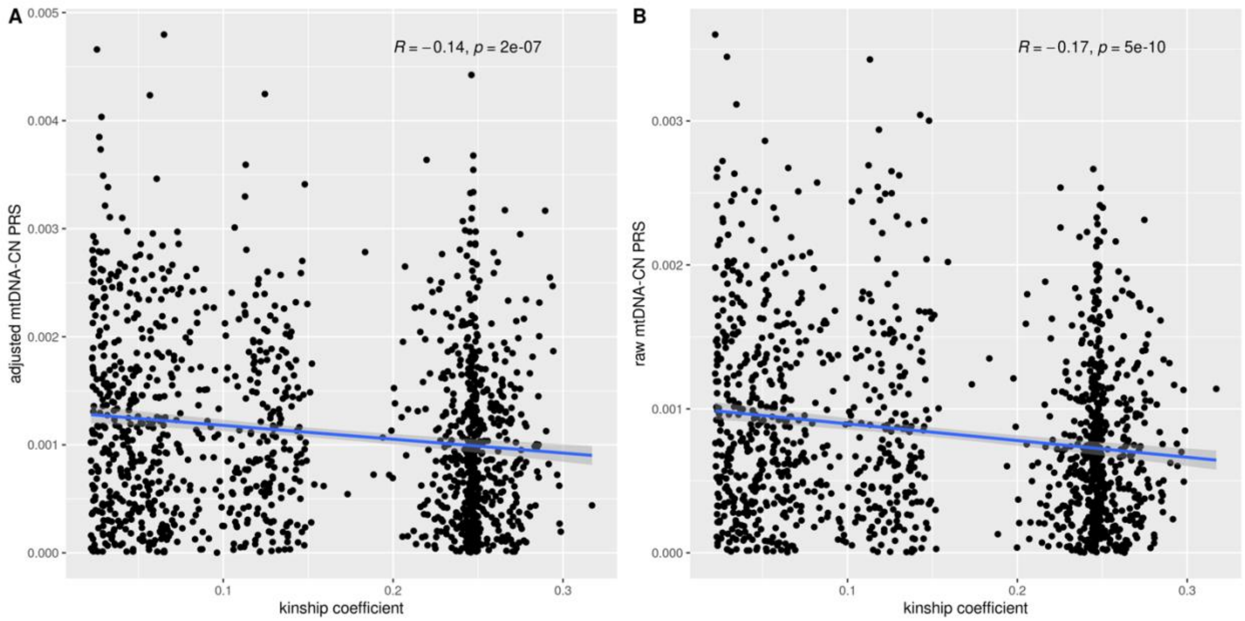

Supplementary Fig. S19 Correlation analysis between mtDNA-CN PRSs and kinship coefficient.

A, scatter plot shows the negative correlation between kinship coefficient and adjusted mtDNA-CN PRS (R = -0.14, p = 2e-07); **B,** scatter plot shows the negative correlation between kinship coefficient and raw mtDNA-CN PRS (R = -0.17, p = 5e-10).

Supplementary Table 1. Association test results of raw blood mtDNA-CN with PD variables in each cohort.

cluster	study	PD diagnosis					MDS UPDRS III					ADL					UPSIT					MoCA				
		sample size	Estimate	Std. Error	t value	Pr(>|t|)	sample size	Estimate	Std. Error	t value	Pr(>|t|)	sample size	Estimate	Std. Error	t value	Pr(>|t|)	sample size	Estimate	Std. Error	t value	Pr(>|t|)	sample size	Estimate	Std. Error	t value	Pr(>|t|)
1	BioFIND	169	-0.02	0.032	-0.72	0.47	172	0.001	0.0009	1.05	0.29	99	0.002	0.002	0.78	0.45	NA	NA	NA	NA	NA	172	0.006	0.008	0.74	0.46
	PDBP	1151	-0.03	0.013	-2.65	0.008	1250	-0.001	0.0003	-3.44	0.0006	1092	0.002	0.000	3.46	0.0006	1069	0.002	0.001	2.59	0.01	1240	0.002	0.002	1.35	0.18
	PPMI	678	-0.02	0.018	-0.83	0.40	676	-0.003	0.0007	-4.00	7.92E-05	515	0.004	0.001	3.59	0.0003	638	0.002	0.001	2.40	0.02	707	0.013	0.004	3.25	0.001
2	HBS	1126	-0.08	0.033	-2.44	0.01	NA	NA	NA	NA	NA	NA	NA	NA	NA	NA	NA	NA	NA	NA	NA	NA	NA	NA	NA	NA
	PDBP	195	0.01	0.035	0.21	0.83	225	-0.003	0.0008	-3.40	0.0008	214	0.002	0.001	2.25	0.03	174	-0.002	0.002	-0.78	0.44	223	-0.001	0.004	-0.19	0.85
	PPMI	854	-0.10	0.019	-5.46	7.18E-08	929	-0.003	0.0006	-4.99	8.80E-07	897	0.002	0.001	3.08	0.003	897	0.005	0.001	5.15	3.70E-07	927	0.009	0.003	3.23	0.001
	STEADY	NA	NA	NA	NA	NA	289	-0.004	0.0019	-2.13	0.03	204	-0.001	0.002	-0.38	0.70	NA	NA	NA	NA	NA	328	0.002	0.010	0.16	0.88
	SURE	NA	NA	NA	NA	NA	258	-0.002	0.0014	-1.36	0.18	257	0.001	0.002	0.27	0.79	NA	NA	NA	NA	NA	257	-0.006	0.007	-0.85	0.41

The table shows the PD-related variables, sample sizes, effect sizes, standard errors, t values, and p values for the output of multivariable regression models, modeling raw blood mtDNA-CN on PD variable + age + sex + PC1-5. MDS UPDRS = Movement Disorder Society-sponsored revision of the Unified Parkinson's Disease Rating Scale; ADL = activities of daily living; UPSIT = University of Pennsylvania Smell Identification Test; MoCA = Montreal Cognitive Assessment.

Supplementary Table S2. Association test results of blood markers with PD variables in PPMI cohort.

PD variables	Neutrophil%					Lymphocyte%					NLR				
	Estimate	Std. Error	t value	Pr(>|t|)	p.adj	Estimate	Std. Error	t value	Pr(>|t|)	p.adj	Estimate	Std. Error	t value	Pr(>|t|)	p.adj
diagnosis	0.018	0.0069	2.64	0.008	0.02	-0.021	0.0072	-2.93	0.004	0.010	0.081	0.0289	2.78	0.005	0.02
MDS_UPDRS_I	0.002	0.0006	4.43	1.26E-05	8.91E-05	-0.002	0.0006	-3.15	0.002	0.007	0.010	0.0024	4.17	5.61E-05	0.0002
MDS_UPDRS_II	0.002	0.0005	4.49	1.27E-05	8.91E-05	-0.003	0.0006	-5.38	1.46E-07	2.04E-06	0.012	0.0022	5.60	1.97E-07	2.75E-06
MDS_UPDRS_III	0.001	0.0002	3.73	0.0002	0.0009	-0.001	0.0002	-4.14	4.89E-05	0.0002	0.004	0.0010	4.24	4.03E-05	0.0002
MDS_UPDRS_IV	0.002	0.0019	1.17	0.24	0.38	-0.002	0.0020	-1.28	0.20	0.30	0.008	0.0079	1.07	0.28	0.44
MoCA	-0.002	0.0010	-2.20	0.03	0.06	0.003	0.0011	2.86	0.005	0.01	-0.011	0.0042	-2.72	0.009	0.02
RBD	0.000	0.0011	0.43	0.67	0.67	0.001	0.0012	0.72	0.47	0.55	-0.001	0.0045	-0.26	0.79	0.79
ESS	0.000	0.0009	0.49	0.62	0.67	-0.001	0.0010	-0.80	0.42	0.54	0.003	0.0037	0.81	0.42	0.53
UPSIT	-0.001	0.0004	-2.62	0.009	0.02	0.001	0.0004	2.53	0.01	0.02	-0.004	0.0016	-2.71	0.007	0.02
ADL	-0.001	0.0003	-4.12	6.15E-05	0.0003	0.001	0.0003	4.35	2.21E-05	0.0002	-0.006	0.0012	-5.05	4.00E-06	2.80E-05
MRI	-0.010	0.0085	-1.23	0.22	0.38	-0.003	0.0091	-0.31	0.76	0.76	-0.014	0.0334	-0.40	0.69	0.74

The table shows the PD-related variables, effect sizes, t values, p values, and false discovery rate (FDR) adjusted p values for the output of multivariable regression models, modeling blood markers on PD variable + age + sex + PC1-5. NLR = neurophil-to-lymphocyte ratio. MDS UPDRS = Movement Disorder Society-Sponsored Revision of the Unified Parkinson's Disease Rating Scale; MoCA = Montreal Cognitive Assessment; RBD = Rapid eye movement sleep behavior disorder; ESS = Epworth Sleepiness Scale; UPSIT = University of Pennsylvania Smell Identification Test; ADL = activities of daily living; MRI = magnetic resonance imaging.

Supplementary Table S3. Association results between blood markers and PD medications.

Medication	Yes/No	Neutrophil-to-lymphocyte Ratio		Neutrophil		Lymphocyte	
		effect size	p value	effect size	p value	effect size	p value
Levodopa	585/163	0.015	0.64	0.006	0.4	-0.003	0.6
Dopamine agonists	398/350	0.081	0.002	0.02	0.0008	-0.013	0.02
Other medications	485/263	-0.01	0.71	-0.006	0.3	-0.001	0.91

The table presents the effect sizes and p-values for the outputs of multivariable regression models: blood marker ~ on levodopa + on dopamine agonists + on other PD medications + age + sex + PC1-5. The reference category for the medications is not taking that specific medication. Only individuals diagnosed with PD who have baseline whole blood bulk RNA-seq and a documented medication history were included in this analysis (N = 748).

Supplementary Table S4. Summary of data source of different traits.

Reference	Phenotype		sample size	population	sex	GWAS Catalog ID	dataset	covariate
Nalls et al. 2019	Parkinson's disease (PD) or proxy PD		482,730 (15,056 cases, 18,618 proxy cases, 449,056 controls)	European	both	GCST009325	3 published studies, 13 new datasets, proxy-case data from the UK BioBank	age, sex, 5PCs
Chong et al. 2022	mtDNA-CN	estimates from SNPchip	383,476	European	both	GCST90026372	UK Biobank	age, age2, sex, chip type, 20PCs, blood cell counts (white blood cell, platelet, and neutrophil counts)
Longcahmps et al. 2021	mtDNA-CN	estimates from WES + SNPchip	440,266	European	both	NA	CHARGE* & UK Biobank	age, sex, PCs, DNA collection site, family structure, cell composition
Gupta et al. 2023	mtDNA-CN	estimates from WGS (adjusted with cell composition)	178,129	AFR,AMR,CSA,EAS,EUR,MID	both	GCST90268497	UK Biobank	age, age*sex, age2, age2*sex, 10PCs, GRM
Gupta et al. 2023	mtDNA-CN	estimates from WGS (raw)	178,129	AFR,AMR,CSA,EAS,EUR,MID	both	GCST90268498	UK Biobank	age, age*sex, age2, age2*sex, 10PCs, GRM

CHARGE – Cohorts for Heart and Aging Research in Genomic Epidemiology

Supplementary Table S5. Correlations between mtDNA-CN PRSs and the actual mtDNA-CN estimates derived from various DNA sources.

cluster	study	sample size	raw mtcn PRS		adj mtcn PRS	
			R	p value	R	p value
1	BioFIND	172	0.036	0.64	0.020	0.80
	PDBP	1258	0.074	0.008	0.056	0.05
	PPMI	716	0.069	0.07	0.101	0.007
2	HBS	1052	0.110	0.0004	0.047	0.13
	LBD	2649	0.091	<0.0001	0.055	0.005
	PDBP	237	0.235	0.0003	0.094	0.15
	PPMI	1088	0.021	0.49	0.101	0.0009
	STEADY-PD3	327	0.225	<0.0001	0.137	0.01
	SURE-PD	258	0.123	0.05	0.126	0.04
3	LBD	1655	-0.001	0.95	-0.003	0.91

The mtDNA-CN estimates underwent transformation using a rank-based inverse normal transform (INT). Clusters 1, 2, and 3 denote platelet-depleted blood samples, platelet-abundant blood samples, and brain samples, respectively.

Supplementary Table S6. MR causal effect estimations between mtDNA-CN and PD.

exposure		outcome		#SNPs	IVW			MR Egger			Weighted median			Weighted mode		
					beta	se	p-value	beta	se	p-value	beta	se	p-value	beta	se	p-value
mtDNA-CN	raw estimates from WGS	PD		57	0.083	0.0813	0.31	0.201	0.1932	0.30	-0.079	0.1222	0.52	-0.230	0.2338	0.33
	adjusted estimates from WGS			31	0.025	0.1059	0.81	0.067	0.2155	0.76	-0.149	0.1436	0.30	-0.200	0.1844	0.29
	estimates from SNPchip			53	0.272	0.1504	0.07	0.116	0.3504	0.74	-0.004	0.2022	0.98	-0.007	0.2560	0.98
	estimates from WES+SNPchip			84	0.066	0.1021	0.52	-0.054	0.2000	0.79	-0.146	0.1562	0.35	-0.127	0.1936	0.51
PD		mtDNA-CN	raw estimates from WGS	49	-0.006	0.0057	0.31	-0.010	0.0127	0.42	-0.003	0.0071	0.72	0.006	0.0139	0.69
			adjusted estimates from WGS	49	-0.007	0.0048	0.14	-0.016	0.0106	0.14	-0.003	0.0074	0.67	0.002	0.0164	0.91
			estimates from SNPchip	43	-0.004	0.003	0.19	-0.016	0.007	0.02	-0.006	0.004	0.16	-0.007	0.008	0.36
			estimates from WES+SNPchip	50	-0.002	0.0033	0.51	-0.008	0.0073	0.31	-0.005	0.0043	0.28	-0.005	0.0063	0.41

The table shows the exposures, outcomes, number of SNPs, effect sizes, standard errors, and p values for the output of MR analysis using inverse variance-weighted (IVW), MR Egger regression, weighted median and weighted mode methods. The results suggest no idrect causal relationship between blood mtDNA-CN and PD risk.

Supplementary Table S7. Sensitivity analysis of MR between mtDNA-CN and PD.

exposure		outcome		heterogeneity test		pleiotropy test (MR Egger)			leave-one-out	Funnel plot
				IVW	MR Egger	intercept	se	p-value		
mtDNA-CN	raw estimates from WGS	PD		0.83	0.82	-0.0050	0.0075	0.50	NULL	symmetric
	adjusted estimates from WGS			0.16	0.14	-0.0023	0.0105	0.82	NULL	symmetric
	estimates from SNPchip			0.09	0.08	0.0041	0.0083	0.62	NULL	symmetric
	estimates from WGS+SNPchip			0.60	0.58	0.0035	0.0050	0.49	NULL	symmetric
PD		mtDNA-CN	raw estimates from WGS	0.006	0.005	0.0007	0.0018	0.69	NULL	symmetric
			adjusted estimates from WGS	0.26	0.26	0.0015	0.0015	0.34	NULL	symmetric
			estimates from SNPchip	0.33	0.34	0.0011	0.0010	0.29	rs35749011	asymmetric
			estimates from WGS+SNPchip	0.01	0.01	0.0009	0.0011	0.41	NULL	symmetric

The table shows the exposures, outcomes, p values of heterogeneity tests, intercepts, standard errors, and p values from pleiotropy test, and outputs from leave-one-out tests and funnel plots.

Supplementary Table S8. Association test results of blood markers with PD variables in UKB.

Blood measurement	Estimate	Std. Error	t value	Pr(>|t|)	P.adj
White blood cell (leukocyte) count	-0.013	0.027	-0.49	0.63	0.64
Platelet count	-3.132	0.893	-3.51	0.0004	0.0006
Plateletcrit	-0.003	0.001	-4.40	<0.0001	<0.0001
Mean platelet (thrombocyte) volume	-0.024	0.017	-1.38	0.17	0.20
Platelet distribution width	-0.004	0.008	-0.47	0.64	0.64
Lymphocyte count	-0.092	0.009	-9.69	<0.0001	<0.0001
Monocyte count	-0.013	0.002	-5.35	<0.0001	<0.0001
Neutrophil count	0.102	0.021	4.86	<0.0001	<0.0001
Eosinophil count	-0.010	0.002	-5.96	<0.0001	<0.0001
Lymphocyte percentage	-1.320	0.119	-11.08	<0.0001	<0.0001
Monocyte percentage	-0.165	0.032	-5.12	<0.0001	<0.0001
Neutrophil percentage	1.626	0.136	12.00	<0.0001	<0.0001
Eosinophil percentage	-0.137	0.023	-6.06	<0.0001	<0.0001
Neutrophil-to-lymphocyte ratio	0.168	0.014	12.37	<0.0001	<0.0001

The table shows the blood variables, effect sizes, standard errors, t values, p values, and false discovery rate (FDR) adjusted p values for the output of multivariable regression models, modeling blood markers on PD diagnosis + age + sex + PC1-5.

Supplementary Table S9. Association test results of blood markers with PD risk in African and multi-ancestry.

Dataset	Blood measurement	African ancestry			Multi-ancestry		
		Estimate	Pr(>|t|)	P.adj	Estimate	Pr(>|t|)	P.adj
AMP PD	Lymphocyte	-0.040	0.17	0.17	-0.021	0.004	0.01
	Neutrophil	0.050	0.06	0.17	0.018	0.008	0.02
	NLR	0.127	0.15	0.17	0.081	0.005	0.02
UK Biobank	White blood cell count	-0.033	0.91	0.91	-0.013	0.63	0.64
	Platelet count	18.774	0.07	0.5	-3.132	0.0004	0.0006
	Plateletcrit	0.015	0.07	0.5	-0.003	<0.0001	<0.0001
	Mean platelet volume	-0.197	0.37	0.6	-0.024	0.17	0.20
	Platelet distribution width	-0.051	0.63	0.68	-0.004	0.64	0.64
	Lymphocyte count	-0.159	0.20	0.6	-0.092	<0.0001	<0.0001
	Monocyte count	-0.020	0.47	0.65	-0.013	<0.0001	<0.0001
	Neutrophil count	0.128	0.52	0.66	0.102	<0.0001	<0.0001
	Eosinophil count	-0.016	0.38	0.6	-0.010	<0.0001	<0.0001
	Lymphocyte%	-1.960	0.27	0.6	-1.320	<0.0001	<0.0001
	Monocyte%	-0.241	0.57	0.66	-0.165	<0.0001	<0.0001
	Neutrophil%	2.330	0.23	0.6	1.626	<0.0001	<0.0001
	Eosinophil%	-0.299	0.32	0.6	-0.137	<0.0001	<0.0001
	NLR	0.125	0.28	0.6	0.168	<0.0001	<0.0001

The table shows the blood variables, effect sizes, p values, and false discovery rate (FDR) adjusted p values for the output of multivariable regression models, modeling blood markers on PD diagnosis + age + sex + PC1-5, in both AMP PD and UKB.
